# Dominant negative retinoic acid receptor initiates tumor formation in mice

**DOI:** 10.1186/1476-4598-5-12

**Published:** 2006-03-24

**Authors:** Tara S Kupumbati, Giorgio Cattoretti, Christine Marzan, Eduardo F Farias, Reshma Taneja, Rafael Mira-y-Lopez

**Affiliations:** 1 Department of Medicine, Mount Sinai School of Medicine, One Gustave L. Levy Place, New York, NY 10029, USA; 2 Current address: Medtronic Heart Valves, 1851 E. DeereAvenue, Santa Ana, CA92705, USA; 3 Institute for Cancer Genetics, Columbia University, 1150 St Nicholas Avenue, New York, NY 10032, USA; 4 Department of Molecular, Cell and Developmental Biology, Mount Sinai School of Medicine, One Gustave L. Levy Place, New York, NY 10029, USA

## Abstract

**Background:**

Retinoic acid suppresses cell growth and promotes cell differentiation, and pharmacological retinoic acid receptor (RAR) activation is anti-tumorigenic. This begs the question of whether chronic physiological RAR activation by endogenous retinoids is likewise anti-tumorigenic.

**Results:**

To address this question, we generated transgenic mice in which expression of a ligand binding defective dominant negative RARα (RARαG303E) was under the control of the mouse mammary tumor virus (MMTV) promoter. The transgene was expressed in the lymphoid compartment and in the mammary epithelium. Observation of aging mice revealed that transgenic mice, unlike their wild type littermates, developed B cell lymphomas at high penetrance, with a median latency of 40 weeks. MMTV-RARαG303E lymphomas were high grade Pax-5+, surface H+L Ig negative, CD69+ and BCL6- and cytologically and phenotypically resembled human adult high grade (Burkitt's or lymphoblastic) lymphomas. We postulated that mammary tumors might arise after a long latency period as seen in other transgenic models of breast cancer. We tested this idea by transplanting transgenic epithelium into the cleared fat pads of wild type hosts, thus bypassing lymphomagenesis. At 17 months post-transplantation, a metastatic mammary adenocarcinoma developed in one of four transplanted glands whereas no tumors developed in sixteen of sixteen endogenous glands with wild type epithelium.

**Conclusion:**

These findings suggest that physiological RAR activity may normally suppress B lymphocyte and mammary epithelial cell growth and that global RAR inactivation is sufficient to initiate a stochastic process of tumor development requiring multiple transforming events. Our work makes available to the research community a new animal resource that should prove useful as an experimental model of aggressive sporadic lymphoma in immunologically uncompromised hosts. We anticipate that it may also prove useful as a model of breast cancer.

## Background

Most of the biologic effects of vitamin A are mediated by all-trans retinoic acid (RA) receptors (RARs) encoded by three genes, RARα, β and γ, each of which give rise to multiple receptor isoforms. RARs regulate gene transcription upon heterodimerization with retinoid X (9-cis RA) receptors (RXRs). RARs and RXRs are members of the superfamily of ligand-dependent transcription factors. In the absence of ligand, RXR-RAR represses gene transcription by recruiting corepressors; upon ligand binding, corepressors are displaced and coactivators recruited, resulting in activation of gene transcription [1,2].

RXR-RAR activation regulates cell proliferation, apoptosis, and differentiation, events that figure prominently in cancer [3-8]. Pharmacological RXR-RAR activation prevents or slows down tumor formation in rodent models of breast cancer [9,10]. We wondered whether chronic physiological RAR activation by endogenous retinoids might exert a qualitatively similar effect. In other words, we wondered whether the anti-cancer effect of pharmacological retinoid doses might represent an enhancement of chronically ongoing, physiological RAR effects. We hypothesized that this might be the case and thus that inhibition of physiological RAR activation might prompt tumor formation. Studies showing that the cellular retinol-binding protein I and RARβ2 are frequently epigenetically silenced in cancer lent indirect support to our hypothesis [11-18]. More pertinent support came from studies showing that RARα antisense [19] and cellular RA-binding protein I [20] overexpression induced tumor formation in mice. Finally, the leukemogenic effect of the t(15;17) translocation that juxtaposes the promyelocytic leukemia and RARα genes, resulting in inhibition of the transactivation function of RARα [21], also encouraged us to pursue our hypothesis.

Because both RARα1 and RARβ2 have been implicated in growth suppression [22,23], we considered studying RARα1/RARβ2 double null mice; however, these die shortly after birth [24], precluding this approach. We therefore addressed our hypothesis using a dominant negative RAR transgenic approach [25]. RARα bearing a G303E point mutation in the ligand binding domain (RARαG303E) binds ligand poorly but retains the ability to heterodimerize with RXR; when transfected into RA-sensitive cells, RARαG303E inhibited the transcriptional activity of endogenous RAR by nearly 100% at physiological RA concentrations (1–10 nM) [26]. Moreover, 100-fold greater concentrations of RA were required for RARα activation of a reporter gene when RARαG303E and RARα were expressed at a ~1:1 molar ratio [26]. These data and the potent in vivo biological effects of RARαG303E [27,28] led us to choose it as the dominant negative RAR for this study.

## Results

### RARαG303E transgenic mice

**Figure 1:**
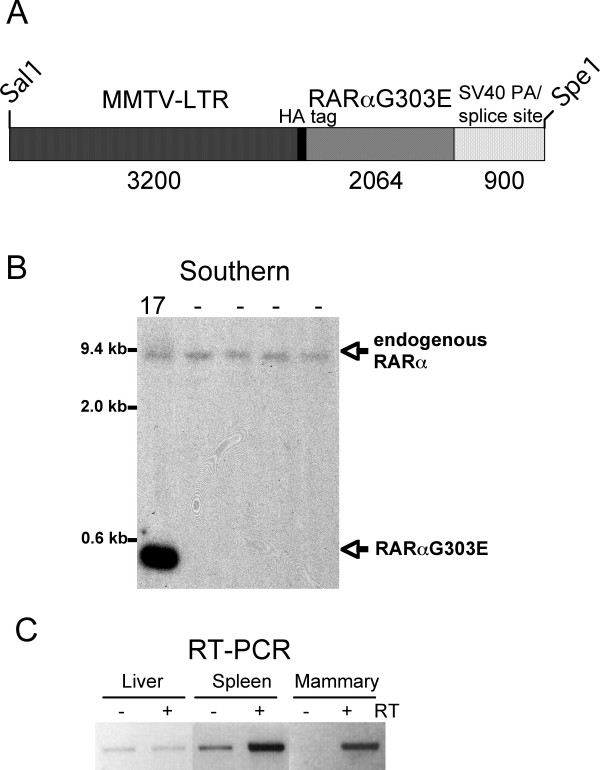
**Tissue transgene expression. (A) **Sketch showing the main features of the transgene construct. **(B) **Southern blot analysis showing the relative amounts of transgene DNA relative to WT RARα DNA in founder 17. Empty lanes correspond to mice that did not integrate the transgene. **(C) **RT-PCR analysis showing transgene expression in the spleen and mammary gland of a young, disease-free line 17 TG female (liver as negative control). Signal detected when the reverse-transcription step was omitted (-RT) is due to contaminating DNA (see Fig. 2 for DNAse I treatment control).

We generated transgenic mice in which the mouse mammary tumor virus (MMTV) long terminal repeat (LTR) drives the expression of the N-terminally HA-tagged RARαG303E dominant negative mutant developed by Saitou et al. [26] (Fig. 1A). The MMTV-LTR has been widely used to target transgene expression to the mammary epithelium and was chosen in this study because of our particular interest in breast cancer. However, the MMTV-LTR is also known to target transgenes to lymphocytes [29-31]. Four female founder mice (#s 17, 24, 6, 39) were obtained as determined by Southern blotting (Fig. 1B shows data for founder 17) or PCR. A hemizygous line was successfully established from founder 17 and maintained over eight generations. The data herein derived from the study of this line and to a lesser extent founder 24 (see Methods for information on founders 6 and 39). We evaluated transgene expression by RT-PCR in mammary tissue, spleen and liver from a transgenic female, taking advantage of the transgene's HA tag to distinguish it from endogenous RARα. Expression was readily detected in both the mammary gland and the spleen but not the liver (Fig. 1C), consistent with previous experience as related above.

### Tumor formation

**Figure 2:**
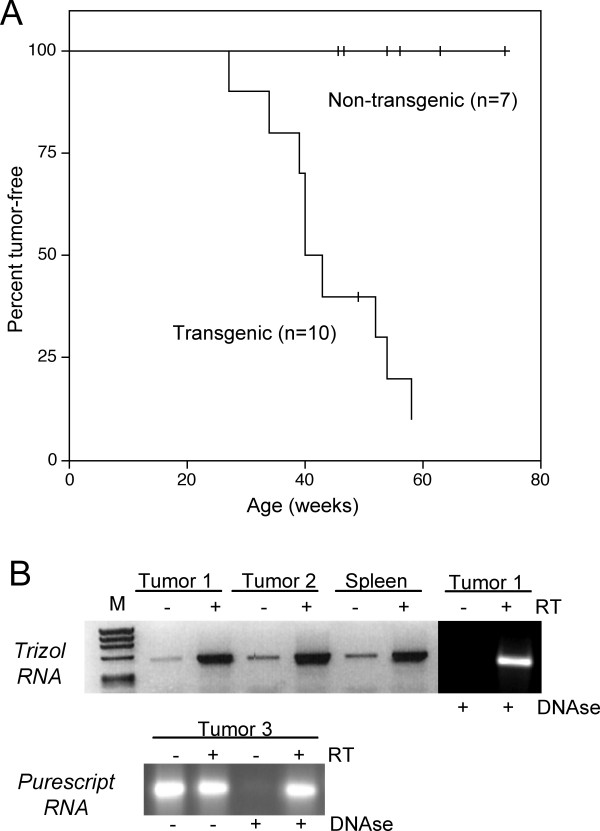
**Tumor incidence and transgene expression. (A) **Kaplan-Meier plot showing the percentage of tumor-free animals as a function of post-natal age in weeks for line 17 TG and WT females.**(B) **Top, tumor transgene expression (RT-PCR using Trizol-isolated RNA). Data is shown for two tumors and spleen of one lymphomatous TG female (M, markers). The relatively low -RT signal is due to contaminating DNA as demonstrated by loss of the signal after DNAse I treatment (last two lanes). Bottom, transgene expression in a tumor from another TG female, using RNA isolated with the less stringent Purescript protocol. The strong -RT signal is lost upon DNAse I treatment.

Based on the transgene expression pattern, we hypothesized that MMTV-RARαG303E mice might be susceptible to lymphoma and/or mammary tumor development. Adult transgenic (TG) female mice (line 17) and wild type (WT) female littermates were examined over time for evidence of tumor development. We found that TG mice developed node-based tumors at high penetrance, with a median latency of 40 weeks; no tumors arose among WT littermates (Fig. 2A). Histological and molecular analyses established that the tumors were B cell lymphomas (below) and RT-PCR analysis demonstrated that they expressed the transgene (Fig. 2B). Founder 24 developed B cell lymphoma at 33 weeks of age. Thus, lymphoma development was associated with MMTV-RARαG303E expression and occurred in two independent TG lines against a tumor-free background in WT mice, suggesting that it is a product of transgene expression rather than insertional mutagenesis. Lymphomagenesis in line 17 was a reproducible feature over 8 generations. Notably, we did not detect mammary tumors, possibly because the latency period for their formation is longer (see below).

### Molecular analysis of tumors

**Figure 3:**
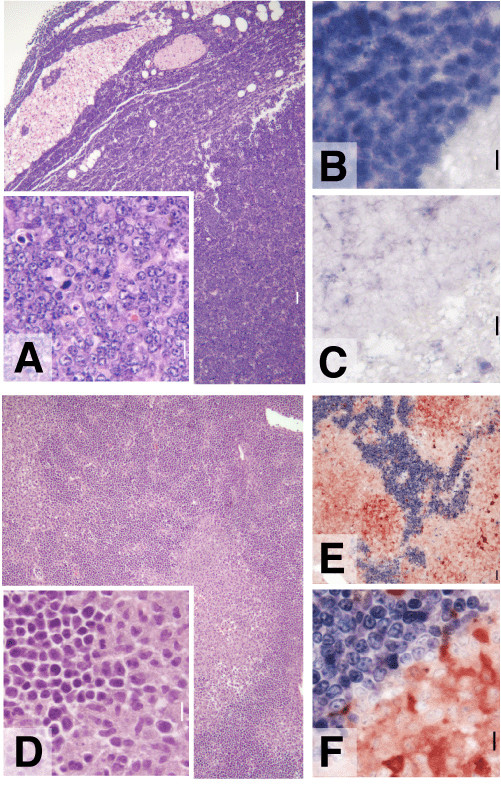
**Histological and immunostaining analysis. (A) **Low power (10x) and high power (40x, inset) image of an H&E stained representative case of MMTV-RARαG303E Burkitt's lymphoma. **(B) **The tumor cells stain for PAX-5 (blue). **(C) **BCL6 staining is negative. **(D) **Low power (10x) and high power (40x, inset) image of an H&E stained biphenotypic B-myelomonocytic tumor. **(E, F) **Low power (E, 10x) and high power (F, 40x) image of Pax-5 (blue) and Mac-2 (red) immunostaining showing separate cell populations (same tumor as in D).

**Figure 4:**
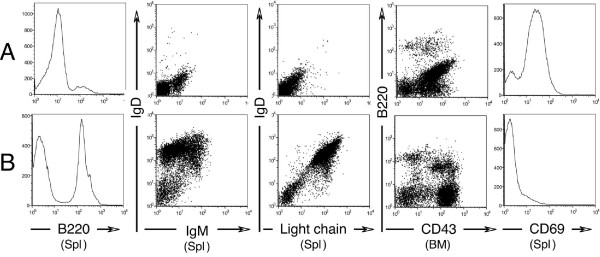
**Flow cytometric analysis. **(A) One representative tumor and (B) spleen (Spl) or bone marrow (BM) from a healthy littermate were analyzed for surface marker expression. Note, in the tumor, the intermediate expression of B220, the absence of surface IgM, IgD and Ig light chains, the coexpression of CD43 and B220 and the positivity for CD69.

All lymphomas were high grade B cell lymphomas, predominantly node-based, and characterized by organ infiltration in liver, spinal cord, and muscle. The morphology ranged from immature blasts with small nucleolus, fine chromatin, mild or moderate nuclear pleomorphism, scanty cytoplasm, abundant mitotic activity (Fig. 3, panel A) to slightly larger cells with vesicular chromatin, prominent nucleolus, mild nuclear pleomorphism and often starry sky appearance, this latter morphology consistent with Burkitt's lymphoma [32]. Consistently, lymphomas were Pax5+, B220+, CD43+, AA41+, CD69+ and negative for surface heavy or light chain immunoglobulins, CD5, CD4, CD8, CD23, CD21, BCL6 (Fig. 3, panels B and C, and Fig. 4). In five typical cases, tumor cells were negative for c-MYC, BCL2, OCT-2, p53 and IRF4. In three typical cases tested, tumor cells were negative for MCL-1. The lymphomas that arose in founder 24 were histologically indistinguishable from typical line 17 lymphomas and like these were Pax5+ and BCL6- by immunostaining. One case had a biphasic cellular infiltrate in the spleen, composed of immature lymphoblasts with fine chromatin and monocytoid immature elements, in adjacent separate areas (Fig. 3, panel D). This tumor was composed of immature lymphoma/leukemia (Pax5+, TdT+, CD3-, Mac2-) and myelomonocytic leukemia (Pax5-, TdT-, CD3-, Mac2+) (Fig. 3, panels E and F) [33]. On secondary transplant, only the B cell component grew.

### Lymphoid system in young transgenic mice is normal

Bone marrow, spleen, thymus and peritoneal cells from 3 TG and 3 WT littermates, age 17 weeks, were analyzed for immature (B220dim CD43+) and mature (B220+ CD43-) bone marrow (BM) B cells, splenic follicular (B220+, IgM+, IgD+, CD23^hi^, CD21^low^), immature (B220+, IgM^low^, IgD-, CD23^low^, CD21^low^, CD43+) and marginal B cells (B220+, IgM+, IgD-, CD23^low^, CD21^hi^), peritoneal B1 (CD5+, IgM+) and B2 cells (CD5-, IgM+), splenic mature T cells (CD4, CD8), thymic immature T cell subsets (CD4, CD8) and activated B cells (CD69). There was no statistically significant difference between WT and TG littermates, with the exception of BM B220dim, CD43+ immature B cells (WT 22 ± 3% versus TG 15 ± 1%, p = 0.035). CD69 mean fluorescence intensity on BM and spleen B cells was statistically not significantly different. Histological analysis did not show lymphoid abnormalities. These results indicate that B cell lymphomas in our model develop without a prodromic pro-B cell hyperplasia.

### Mammary tumor formation upon epithelial transplantation

**Figure 5:**
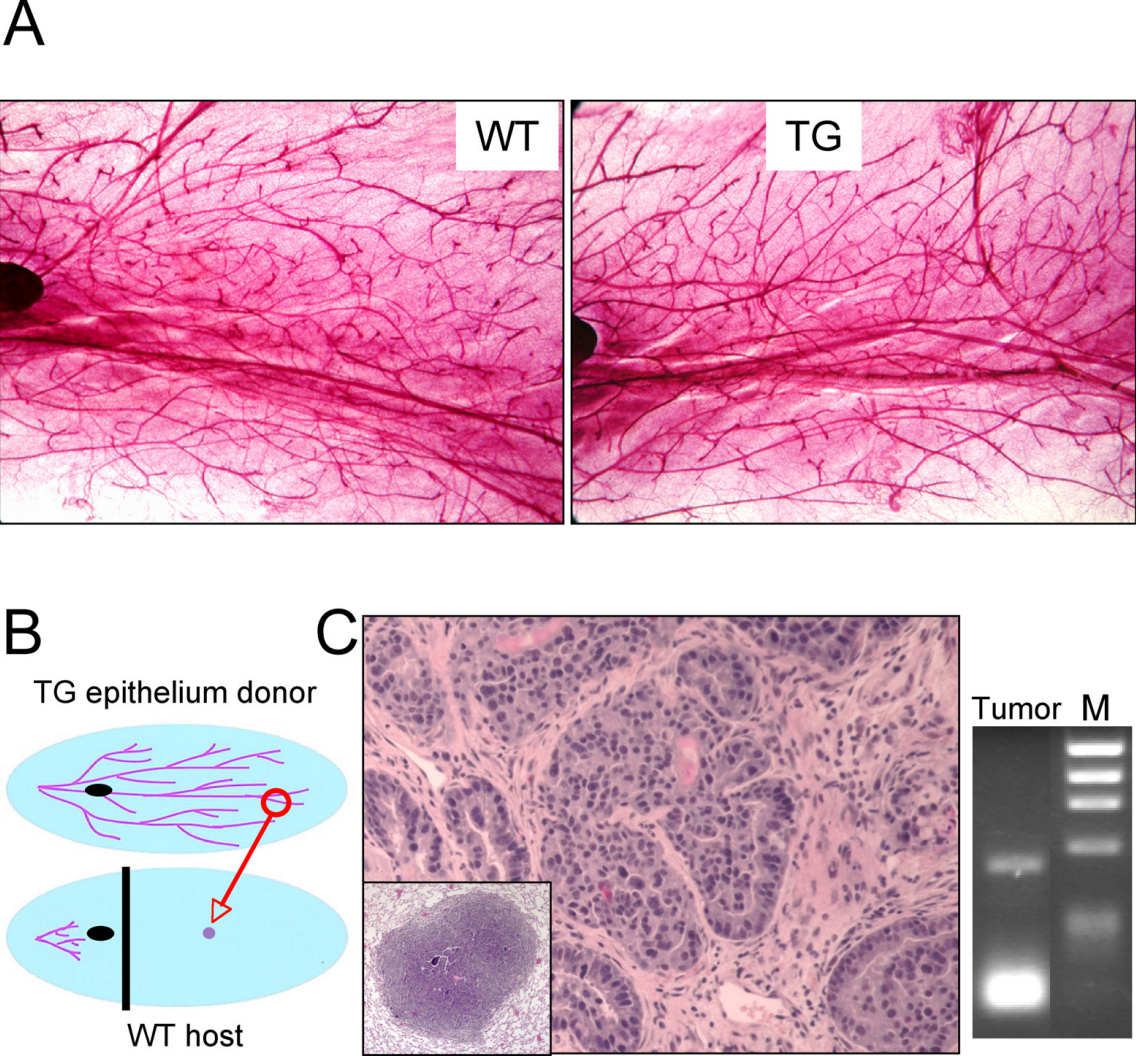
**Mammary epithelial transplantation. (A) **Abdominal mammary gland whole mounts showing mature mammary duct development in 17-week old TG and WT virgin females. The mammary lymph node, a positional landmark, is partially visible (darkly stained body, left end of panel). **(B) **Diagram illustrating the procedure of mammary epithelial transplantation. Blue oval, mammary fat pad; the ductal tree is represented in purple; small black oval, mammary lymph node; tissue to the left of the vertical line is surgically removed leaving a cleared fat pad. See Methods for details. **(C) **Left, H&E stained section of the mammary adenocarcinoma and metastatic lung nodule (inset) that arose after transplantation of TG epithelium into the cleared fat pad of a WT host. Tissue was fixed in formalin and embedded in paraffin. Right, DNA was isolated from parallel sections and PCR performed, confirming the presence of the transgene in the tumor (M, markers).

Whole mount analysis of mammary glands taken from young sexually mature females showed that ductal development was complete in both TG and WT animals, with the ductal tree reaching the confines of the fat pad (Fig. 5A). A similar finding was made by Costa et al. in a related dominant negative RAR transgenic model [34]. Therefore, the fact that we did not observed mammary tumor development within the time scale shown in Fig. 2A cannot be attributed to altered ductal development, nor to our choice of the FVB/N strain since MMTV-driven expression of "classical" oncogenes and other genes is tumorigenic in this strain. In several of these models, some or most tumors do not develop until late in the life of the animal [35-37]. We therefore wondered whether mammary tumors might arise in MMTV-RARαG303E mice were it not for the fact that they succumb to lymphomas first. To begin to test this idea, we performed an experiment designed to bypass lymphoma development. We transplanted TG mammary epithelium from young disease-free females to a WT host whose abdominal mammary glands had been precleared of native epithelium (Fig. 5B). Sham controls confirmed that the endogenous epithelium was fully removed. A total of four transplant recipients were allowed to mate freely and followed over time. Two mice died disease-free and were lost to follow-up. One of the transplanted glands in the remaining two subjects developed a mammary adenocarcinoma with lung metastasis 17 months after transplantation (Fig. 5C). Neither mouse developed lymphoma. In total, a metastatic mammary carcinoma arose in one of four glands transplanted with TG epithelium but in none of sixteen host glands bearing intact WT epithelium. This result was not statistically significant (chi square), which is hardly surprising given the very preliminary nature of the study. However, we were able to document that the mammary carcinoma was transgenic (Fig. 5C), as expected from the fact that *i) *it arose in a gland that received a TG epithelium transplant and *ii) *sham controls invariably confirmed that clearing of endogenous WT epithelium was complete. Thus, there is virtually no doubt that the mammary epithelium is susceptible to transformation by RARαG303E. This and the success of our strategy in bypassing lymphoma development suggest that further studies of the susceptibility of MMTV-RARαG303E mice to mammary tumor formation, particularly in combination with other transforming events, is warranted. It will also be worthwhile to test for evidence of mammary hyperplasia in TG versus WT littermates at an early age, i.e., prior to the time when lymphomas begin to appear. As we pursue these studies, it will be of interest to test whether similar proproliferative and antiapoptotic strategies underlie dominant negative RAR transformation of B cells and mammary epithelial cells. For instance, decreased cyclin D1, cdk4 and cyclin E are associated with RA mediated G1 arrest [38].

## Discussion

Lymphomas in our model were transitional B cell type because of the presence of markers of immaturity (CD43, lack of surface Ig, occasional TdT positivity). They also lacked BCL6, which is otherwise typically present in human germinal center-derived lymphomas, including high grade human Burkitt's [39]. We did not find T cell lymphomas or frank myeloid leukemias. The interesting finding of a mixed B and myelomonocytic tumor in one mouse (Results section) suggests origination of the process in a progenitor retaining the plasticity to be programmed in two usually distinct lineages [40].

The growth of lymphomas is typically supported by the expression of an anti-apoptotic protein [39,41,42]; however, MMTV-RARαG303E lymphomas did not express BCL6, BCL2 or MCL1 (Results section), and preliminary data indicated that BCL-XL is weakly expressed. These findings suggest that a survival mechanism that is independent of these potent inhibitors of apoptosis wards off cell death in our model.

Despite the fact that they do not overexpress c-MYC protein, MMTV-RARαG303E lymphomas resembled, histologically and phenotypically, other types of high grade lymphomas with transitional phenotype, such as the ones driven by deregulated MYC expression [43-46]. However, in the latter models, most mice succumb to lymphomas at 14–30 weeks of life whereas in our model morbidity began later (27 weeks), suggesting a requirement for additional transforming events. Another difference was the absence of BM pro-B cell hyperplasia and activation, noticed in the Eμ-MYC mice [44]. In fact, healthy MMTV-RARαG303E mice had moderately reduced numbers of pro-B cells in the bone marrow and lacked CD69.

Interestingly, Manshouri et al. generated RARα antisense transgenic mice and showed that they develop B and T cell lymphomas [19]. However, whereas in this model tumor development was associated with high BCL2 protein expression [47], our model generated exclusively BCL2 negative B cell lymphomas restricted to a specific window of differentiation, with a homogeneous transitional phenotype, all of high grade. The absence of immunologic abnormalities preceding the lymphoma onset is also a unique aspect of our model. These differences may be explained by differences in experimental design with respect both to the nature of the transgene (RARαG303E as opposed to RARα antisense) and the promoter driving its expression (MMTV-LTR as opposed to the pMAMneo expression vector which confers wider tissue expression).

Though the evidence is very preliminary at this stage, our observation that RARαG303E has the potential to transform the mammary epithelium is intriguing. A large scale version of our pilot study is required to determine the magnitude of this potential. Wang et al. recently developed a transgenic model virtually identical to ours (below) and reported increased mammary branching in pubertal transgenic females [48], an observation that may or may not relate to our finding. It is important to note that our observation of mammary transformation by a dominant negative RAR is not without precedent. Thus, early studies by Berard et al. showed that lung and mammary carcinomas develop in MMTV-mRARβ4-like transgenic mice [49]. However, whether this can be attributed to a dominant negative-like activity of mRARβ4 [50] remains unclear since the transcriptional activity of this receptor is promoter context- and response element-dependent [51] and since the altered N-terminus of the mRARβ4-like transgene could conceivably have introduced an artifact. However, the use of RARαG303E is also not without caveat; thus, in K14-RARαG303E transgenic mice, which exhibit a skin phenotype, RARαG303E was shown to act via a pathway that is independent of DNA binding [52].

While this manuscript was in preparation, Wang et al. reported the generation of an apparently identical model to that we have described here, i.e., transgenic MMTV-RARαG303E mice in an FVB/N background [53]. Their mice developed B cell lymphomas with similar kinetics and the same phenotype as we have reported here. All seven transgenic founders they obtained developed B cell lymphoma, thus leaving little doubt that this phenotype is a result of transgene expression per se and not insertional mutagenesis. Our study and that of Wang et al. provide mutual and independent confirmation of the susceptibility of MMTV-RARαG303E mice to the formation of immature B cell lymphomas. Importantly, their study showed that RA treatment inhibited lymphoma growth, an observation that strongly suggests that RARαG303E acted as a bonafide dominant negative RAR. Our study extends theirs in several important regards: *i) *we demonstrate through extensive analysis that the transgene does not affect pre-tumor lymphoid differentiation (Results section); *ii) *we show by immunostaining that the tumors were positive for Pax-5, which is the most faithful and restricted (in the hematopoietic system) lymphoid transcription factor and remarkably evolutionarily conserved in B cells (Fig. 3); *iii) *we immunostained tumors for a number of relevant proteins including c-MYC, p53, BCL6, BCL2 and MCL1 (Fig. 3 and Results section); *iv) *we describe an unusual biphenotypic B-myelomonocytic tumor that implies origination of the transformation process in a plastic progenitor cell (Fig. 3 and Discussion); and *v) *we provide very early but nevertheless intriguing evidence that RARαG303E can transform the mammary epithelium (Fig. 5).

Conclusion

Our data suggest that perturbation of physiological RAR activity has major health consequences. Thus, expression of a dominant negative RAR in mouse lymphoid tissue and mammary epithelium resulted in their malignant transformation. This is of potential clinical significance in view of the growing body of evidence showing that epigenetic changes underlie many human malignancies and specifically that epigenetic silencing of the cellular retinol binding protein I and RARβ2 are common events in human lymphoma and breast cancer.

## Methods

### Generation and maintenance of transgenic mice

All experiments were performed under Institutional Animal Care and Use Committee approval. RARαG303E was excised from pCMX-RAR-E [26] as a Hind III-Bam HI fragment and Klenow filled to generate blunt ends. This fragment was subcloned into MMTV-SV40-Bssk [54] digested with EcoRI and Klenow filled (blunt end ligation). Insert orientation was determined by DNA sequencing. The entire MMTV promoter-RARαG303E fragment was obtained by digestion with Sal I and Spe I (see Fig. 1A). This fragment was gel eluted and purified by ultracentrifugation through CsCl gradient and turned over to the Mouse Genetics Shared Research Facility for pronuclear microinjection (FVB/N background). The data herein was obtained from the study of a hemizygous line established from founder 17; founder 24 died of B cell lymphoma and its tumors were also characterized. Founder 6 transmitted the transgene to its offspring but a hemizygous line was not successfully maintained. Founder 39 died of unknown cause. Line 17 TG males were sterile as judged by their inability to sire litters after multiple attempts. Male sterility was previously described and studied in a related dominant negative RAR model [34] and was likewise observed by Wang et al. [48]. MMTV line 17 was maintained by interbreeding TG females to WT males; the transgene was transmitted in a Mendelian fashion.

### Genotyping and RT-PCR

Tail DNA was digested with KpnI and SacI to release a 503 bp RARαG303E fragment and Southerns probed with RARαG303E cDNA. Endogenous RARα yielded a ~9 kb fragment. PCR genotyping as well as RT-PCR took advantage of the HA tag to distinguish the transgene from WT RARα. For PCR, amplification (25 cycles) was carried out with forward primer (5'-3') TACCCCTACGACGTGCCCGACTATGCCAGC, reverse primer (5'-3') GTTTCTCACAGACTCCTTGGACATGCCCAC, and 66°C annealing temperature. Total RNA was isolated using either the classical, more stringent Trizol method [55] or the Purescript kit from Gentra Systems (Minneapolis, MN). For RT-PCR, 2 μg of total RNA were reverse transcribed using random primers and 1/10^th ^of the cDNA used as PCR input. Amplification was carried out with the same primers and conditions as above. Amplification reactions in which the RT step was omitted were included to control for contaminating DNA.

### Flow cytometric analysis

Single cell suspensions from normal organs or tumor mass were stained with the following antibody combinations: A: B220, IgM, IgD, Ig kappa light chain.; B: B220, CD23, CD21, CD11b.; C: IgM, CD43, CD5, CD19; D: B220, CD69, CD80.; E: B220, A44.1, IgM, CD43.; F: CD3, CD4, CD8. All antibodies except for IgD-Pe (Southern Biotechnology Inc. Birmingham, ALA) were form BD-Pharmingen (San Diego, CA). A Becton Dickinson FACS Calibur (BD-Pharmingen) was used for the analysis. FlowJo 6.1.1 (Tree Star Inc. Ashland, OR) was used for the analysis and graphic elaboration of the data.

### Histology, immunohistochemistry and immunofluorescence

Four μm-thick formalin fixed, paraffin embedded sections were stained for H&E or immunostained as published previously [56,57]. Briefly, dewaxed, 0.01 mM EDTA antigen-retrieved sections were blocked in 5% defatted milk powder in TBS-TritonX100, incubated overnight with primary antibodies against: rabbit anti BCL6, c-MYC, Bcl2, OCT-2, and IRF4 (Santa Cruz Biotechnology, CA), mouse anti Pax5, rat anti B220, CD138 (BD-Pharmingen), PNA lectin (Vector, Burlingame, CA) and rat anti Mac-2 (Cedarlane, Ontario, Canada), p53 CM5 (Novocastra, Newcastle-U-Tyne, UK). The specificity and reactivity of these reagents on mouse tissue was previously defined. Two rabbit antibodies against MCL-1 (S-19, sc-819 from Santa Cruz and 600-401-394 from Rockland, Gilbertsville, PA), generated against two unrelated epitopes of the molecule, were used to stain tumor tissue. These antibodies reacted with mouse normal germinal center and other selected tissues in an identical manner; the former was used at 0.2 μg/ml for further characterization of the lymphomas. The primary antibodies were counterstained with biotin- or Alkaline Phosphatase (AP) -conjugated secondary antibodies (Vector or Southern Biotechnology Associates, Birmingham, ALA). Biotin-conjugated antibodies received either Avidin-HRP (DAKOUSA, Carpintera, CA) or Avidin-AP. The enzymes were developed with AEC (AminoethylCarbazole, Sigma) or NBT-BCIP (Roche). Double immunohistochemical staining was performed as published previously [58] with non-crossreacting combinations of primary and secondary antibodies.

### Mammary gland procedures

The technique of mammary epithelial transplantation has been described in detail [59]. Briefly, a 14-week old RARαG303E female (donor) and 3 to 4-week old virgin WT females (recipients) were anesthetized, the abdominal mammary glands of the donor surgically exposed, and 2 mm^3 ^gland fragments excised and placed on sterile PBS on ice. The abdominal mammary glands of the recipients were then surgically exposed and cleared of epithelium by cauterizing the fat pad at a point distal to the mammary lymph node (in 3 to 4-week old virgins the ducts lie proximal to the node, Fig. 5B); two donor fragments were implanted into a pocket prepared in the middle of each cleared fat pad. Finally, skin flaps were sutured with wound clips. Sham controls underwent gland clearing without receiving epithelial implants. Mammary whole mounts were prepared as described [60].

## Abbreviations

RAR, retinoic acid receptor

RARαG303E, dominant negative RAR

RXR, retinoid X receptor

MMTV, mouse mammary tumor virus

LTR, long terminal repeat

HA, hemagglutinin

TG, transgenic

WT, wild type

Ig, immunoglobulin

H, heavy Ig chain

I, light Ig chain

BM, bone marrow

PCR, polymerase chain reaction

RT-PCR, reverse transcription PCR

AP, alkaline phosphatase

K14, keratin 14

PBS, phosphate buffered saline

## Competing interests

The author(s) declare that they have no competing interests.

## Authors' contributions

TSK synthesized and purified the MMTV-RARαG303E construct, designed the protocols for genotyping and RT-PCR, identified and bred the founder mice, generated the data in Figs. 1B and 5A, and contributed in numerous other ways. GC was responsible for all the expert and extensive molecular marker analyses of WT and TG lymphoid tissues (Figs. 3, 4, text and data not shown), and wrote the corresponding parts of the manuscript. CM generated the data in Figs. 1C, 2B and 5C. EFF performed the mammary gland transplantation experiments (Fig. 5B). RT contributed intellectually to all aspects of experimental design and interpretation. RML conceived and supervised the study, generated the data in Fig. 2A, and wrote the manuscript. All authors read and approved the final manuscript.

## References

[B1] Chambon P (1996). A decade of molecular biology of retinoic acid receptors. Faseb J.

[B2] Mangelsdorf DJ, Kliewer SA, Kakizuka A, Umesono K, Evans RM (1993). Retinoid receptors. Recent Prog Horm Res.

[B3] Seewaldt VL, Johnson BS, Parker MB, Collins SJ, Swisshelm K (1995). Expression of retinoic acid receptor beta mediates retinoic acid-induced growth arrest and apoptosis in breast cancer cells. Cell Growth Differ.

[B4] Seewaldt VL, Caldwell LE, Johnson BS, Swisshelm K, Collins SJ, Tsai S (1997). Inhibition of retinoic acid receptor function in normal human mammary epithelial cells results in increased cellular proliferation and inhibits the formation of a polarized epithelium in vitro. Exp Cell Res.

[B5] Niles RM (2000). Recent advances in the use of vitamin A (retinoids) in the prevention and treatment of cancer. Nutrition.

[B6] Lonardo F, Dragnev KH, Freemantle SJ, Ma Y, Memoli N, Sekula D, Knauth EA, Beebe JS, Dmitrovsky E (2002). Evidence for the epidermal growth factor receptor as a target for lung cancer prevention. Clin Cancer Res.

[B7] Lee HY, Dohi DF, Kim YH, Walsh GL, Consoli U, Andreeff M, Dawson MI, Hong WK, Kurie JM (1998). All-trans retinoic acid converts E2F into a transcriptional suppressor and inhibits the growth of normal human bronchial epithelial cells through a retinoic acid receptor- dependent signaling pathway. J Clin Invest.

[B8] Hatoum A, El-Sabban ME, Khoury J, Yuspa SH, Darwiche N (2001). Overexpression of retinoic acid receptors alpha and gamma into neoplastic epidermal cells causes retinoic acid-induced growth arrest and apoptosis. Carcinogenesis.

[B9] Anzano MA, Byers SW, Smith JM, Peer CW, Mullen LT, Brown CC, Roberts AB, Sporn MB (1994). Prevention of breast cancer in the rat with 9-cis-retinoic acid as a single agent and in combination with tamoxifen. Cancer Res.

[B10] Wu K, Kim HT, Rodriquez JL, Hilsenbeck SG, Mohsin SK, Xu XC, Lamph WW, Kuhn JG, Green JE, Brown PH (2002). Suppression of mammary tumorigenesis in transgenic mice by the RXR-selective retinoid, LGD1069. Cancer Epidemiol Biomarkers Prev.

[B11] Xu XC, Sneige N, Liu X, Nandagiri R, Lee JJ, Lukmanji F, Hortobagyi G, Lippman SM, Dhingra K, Lotan R (1997). Progressive decrease in nuclear retinoic acid receptor beta messenger RNA level during breast carcinogenesis. Cancer Res.

[B12] Sirchia SM, Ferguson AT, Sironi E, Subramanyan S, Orlandi R, Sukumar S, Sacchi N (2000). Evidence of epigenetic changes affecting the chromatin state of the retinoic acid receptor beta2 promoter in breast cancer cells. Oncogene.

[B13] Widschwendter M, Berger J, Daxenbichler G, Muller-Holzner E, Widschwendter A, Mayr A, Marth C, Zeimet AG (1997). Loss of retinoic acid receptor beta expression in breast cancer and morphologically normal adjacent tissue but not in the normal breast tissue distant from the cancer. Cancer Res.

[B14] Widschwendter M, Berger J, Hermann M, Muller HM, Amberger A, Zeschnigk M, Widschwendter A, Abendstein B, Zeimet AG, Daxenbichler G, Marth C (2000). Methylation and silencing of the retinoic acid receptor-beta2 gene in breast cancer. J Natl Cancer Inst.

[B15] Bean GR, Scott V, Yee L, Ratliff-Daniel B, Troch MM, Seo P, Bowie ML, Marcom PK, Slade J, Kimler BF, Fabian CJ, Zalles CM, Broadwater G, Baker JCJ, Wilke LG, Seewaldt VL (2005). Retinoic acid receptor-beta2 promoter methylation in random periareolar fine needle aspiration. Cancer Epidemiol Biomarkers Prev.

[B16] Kuppumbatti YS, Bleiweiss IJ, Mandeli JP, Waxman S, Mira-y-Lopez R (2000). Cellular retinol-binding protein expression and breast cancer. J Natl Cancer Inst.

[B17] Arapshian A, Bertran S, Kuppumbatti YS, Nakajo S, Mira-y-Lopez R (2004). Epigenetic CRBP downregulation appears to be an evolutionarily conserved (human and mouse) and oncogene-specific phenomenon in breast cancer. Mol Cancer.

[B18] Esteller M, Guo M, Moreno V, Peinado MA, Capella G, Galm O, Baylin SB, Herman JG (2002). Hypermethylation-associated Inactivation of the Cellular Retinol-Binding-Protein 1 Gene in Human Cancer. Cancer Res.

[B19] Manshouri T, Yang Y, Lin H, Stass SA, Glassman AB, Keating MJ, Albitar M (1997). Downregulation of RAR alpha in mice by antisense transgene leads to a compensatory increase in RAR beta and RAR gamma and development of lymphoma. Blood.

[B20] Perez-Castro AV, Tran VT, Nguyen-Huu MC (1993). Defective lens fiber differentiation and pancreatic tumorigenesis caused by ectopic expression of the cellular retinoic acid-binding protein I. Development.

[B21] Zelent A, Guidez F, Melnick A, Waxman S, Licht JD (2001). Translocations of the RARalpha gene in acute promyelocytic leukemia. Oncogene.

[B22] Fitzgerald P, Teng M, Chandraratna RA, Heyman RA, Allegretto EA (1997). Retinoic acid receptor alpha expression correlates with retinoid-induced growth inhibition of human breast cancer cells regardless of estrogen receptor status. Cancer Res.

[B23] Swisshelm K, Ryan K, Lee X, Tsou HC, Peacocke M, Sager R (1994). Down-regulation of retinoic acid receptor beta in mammary carcinoma cell lines and its up-regulation in senescing normal mammary epithelial cells. Cell Growth Differ.

[B24] Lohnes D, Mark M, Mendelsohn C, Dolle P, Dierich A, Gorry P, Gansmuller A, Chambon P (1994). Function of the retinoic acid receptors (RARs) during development (I). Craniofacial and skeletal abnormalities in RAR double mutants. Development.

[B25] Tsai S, Bartelmez S, Heyman R, Damm K, Evans R, Collins SJ (1992). A mutated retinoic acid receptor-alpha exhibiting dominant-negative activity alters the lineage development of a multipotent hematopoietic cell line. Genes Dev.

[B26] Saitou M, Narumiya S, Kakizuka A (1994). Alteration of a single amino acid residue in retinoic acid receptor causes dominant-negative phenotype. J Biol Chem.

[B27] Saitou M, Sugai S, Tanaka T, Shimouchi K, Fuchs E, Narumiya S, Kakizuka A (1995). Inhibition of skin development by targeted expression of a dominant-negative retinoic acid receptor. Nature.

[B28] Yamaguchi M, Nakamoto M, Honda H, Nakagawa T, Fujita H, Nakamura T, Hirai H, Narumiya S, Kakizuka A (1998). Retardation of skeletal development and cervical abnormalities in transgenic mice expressing a dominant-negative retinoic acid receptor in chondrogenic cells. Proc Natl Acad Sci U S A.

[B29] Leder A, Pattengale PK, Kuo A, Stewart TA, Leder P (1986). Consequences of widespread deregulation of the c-myc gene in transgenic mice: multiple neoplasms and normal development. Cell.

[B30] Hundley JE, Koester SK, Troyer DA, Hilsenbeck SG, Subler MA, Windle JJ (1997). Increased tumor proliferation and genomic instability without decreased apoptosis in MMTV-ras mice deficient in p53. Mol Cell Biol.

[B31] DeRocco SE, Iozzo R, Ma XP, Schwarting R, Peterson D, Calabretta B (1997). Ectopic expression of A-myb in transgenic mice causes follicular hyperplasia and enhanced B lymphocyte proliferation. Proc Natl Acad Sci U S A.

[B32] Morse HC, Anver MR, Fredrickson TN, Haines DC, Harris AW, Harris NL, Jaffe ES, Kogan SC, MacLennan IC, Pattengale PK, Ward JM (2002). Bethesda proposals for classification of lymphoid neoplasms in mice. Blood.

[B33] Kogan SC, Ward JM, Anver MR, Berman JJ, Brayton C, Cardiff RD, Carter JS, de Coronado S, Downing JR, Fredrickson TN, Haines DC, Harris AW, Harris NL, Hiai H, Jaffe ES, MacLennan IC, Pandolfi PP, Pattengale PK, Perkins AS, Simpson RM, Tuttle MS, Wong JF, Morse HC (2002). Bethesda proposals for classification of nonlymphoid hematopoietic neoplasms in mice. Blood.

[B34] Costa SL, Boekelheide K, Vanderhyden BC, Seth R, McBurney MW (1997). Male infertility caused by epididymal dysfunction in transgenic mice expressing a dominant negative mutation of retinoic acid receptor alpha 1. Biol Reprod.

[B35] White DE, Cardiff RD, Dedhar S, Muller WJ (2001). Mammary epithelial-specific expression of the integrin-linked kinase (ILK) results in the induction of mammary gland hyperplasias and tumors in transgenic mice. Oncogene.

[B36] Wechselberger C, Strizzi L, Kenney N, Hirota M, Sun Y, Ebert A, Orozco O, Bianco C, Khan NI, Wallace-Jones B, Normanno N, Adkins H, Sanicola M, Salomon DS (2005). Human Cripto-1 overexpression in the mouse mammary gland results in the development of hyperplasia and adenocarcinoma. Oncogene.

[B37] Amundadottir LT, Johnson MD, Merlino G, Smith GH, Dickson RB (1995). Synergistic interaction of transforming growth factor alpha and c-myc in mouse mammary and salivary gland tumorigenesis. Cell Growth Differ.

[B38] Seewaldt VL, Kim JH, Caldwell LE, Johnson BS, Swisshelm K, Collins SJ (1997). All-trans-retinoic acid mediates G1 arrest but not apoptosis of normal human mammary epithelial cells. Cell Growth Differ.

[B39] Jaffe ES, Harris AL, Stein H, Vardiman JW, Jaffe ES (2001). Pathology and Genetics of Tumours of the Haematopoietic and Lymphoid Tissues WHO Blue Books.

[B40] Xie H, Ye M, Feng R, Graf T (2004). Stepwise reprogramming of B cells into macrophages. Cell.

[B41] Sanchez-Beato M, Sanchez-Aguilera A, Piris MA (2003). Cell cycle deregulation in B-cell lymphomas. Blood.

[B42] Zhou P, Qian L, Bieszczad CK, Noelle R, Binder M, Levy NB, Craig RW (1998). Mcl-1 in transgenic mice promotes survival in a spectrum of hematopoietic cell types and immortalization in the myeloid lineage. Blood.

[B43] Adams JM, Harris AW, Pinkert CA, Corcoran LM, Alexander WS, Cory S, Palmiter RD, Brinster RL (1985). The c-myc oncogene driven by immunoglobulin enhancers induces lymphoid malignancy in transgenic mice. Nature.

[B44] Langdon WY, Harris AW, Cory S, Adams JM (1986). The c-myc oncogene perturbs B lymphocyte development in E-mu-myc transgenic mice. Cell.

[B45] Harris AW, Pinkert CA, Crawford M, Langdon WY, Brinster RL, Adams JM (1988). The E mu-myc transgenic mouse. A model for high-incidence spontaneous lymphoma and leukemia of early B cells. J Exp Med.

[B46] Kovalchuk AL, Qi CF, Torrey TA, Taddesse-Heath L, Feigenbaum L, Park SS, Gerbitz A, Klobeck G, Hoertnagel K, Polack A, Bornkamm GW, Janz S, Morse HC (2000). Burkitt lymphoma in the mouse. J Exp Med.

[B47] Haidar MA, Manshouri T, Keating MJ, Kantarjian HM, Freireich EJ, Mehta K, Albitar M (2000). Downregulation of the p53 tumor suppressor gene and upregulation of the bcl-2 gene in retinoic acid receptor alpha-deficient transgenic mice. Int J Oncol.

[B48] Wang YA, Shen K, Wang Y, Brooks SC (2005). Retinoic acid signaling is required for proper morphogenesis of mammary gland. Dev Dyn.

[B49] Berard J, Gaboury L, Landers M, De Repentigny Y, Houle B, Kothary R, Bradley WE (1994). Hyperplasia and tumours in lung, breast and other tissues in mice carrying a RAR beta 4-like transgene. Embo J.

[B50] Nagpal S, Zelent A, Chambon P (1992). RAR-beta 4, a retinoic acid receptor isoform is generated from RAR-beta 2 by alternative splicing and usage of a CUG initiator codon. Proc Natl Acad Sci U S A.

[B51] Nagpal S, Saunders M, Kastner P, Durand B, Nakshatri H, Chambon P (1992). Promoter context- and response element-dependent specificity of the transcriptional activation and modulating functions of retinoic acid receptors. Cell.

[B52] Chen CF, Lohnes D (2005). Dominant-negative retinoic acid receptors elicit epidermal defects through a non-canonical pathway. J Biol Chem.

[B53] Wang YA, Shen K, Ishida Y, Wang Y, Kakizuka A, Brooks SC (2005). Induction of murine leukemia and lymphoma by dominant negative retinoic acid receptor alpha. Mol Carcinog.

[B54] Guy CT, Webster MA, Schaller M, Parsons TJ, Cardiff RD, Muller WJ (1992). Expression of the neu protooncogene in the mammary epithelium of transgenic mice induces metastatic disease. Proc Natl Acad Sci U S A.

[B55] Chomczynski P, Sacchi N (1987). Single-step method of RNA isolation by acid guanidinium thiocyanate-phenol-chloroform extraction. Anal Biochem.

[B56] Ye BH, Cattoretti G, Shen Q, Zhang J, Hawe N, de Waard R, Leung C, Nouri-Shirazi M, Orazi A, Chaganti RS, Rothman P, Stall AM, Pandolfi PP, Dalla-Favera R (1997). The BCL-6 proto-oncogene controls germinal-centre formation and Th2-type inflammation. Nat Genet.

[B57] Cattoretti G, Chang CC, Cechova K, Zhang J, Ye BH, Falini B, Louie DC, Offit K, Chaganti RS, Dalla-Favera R (1995). BCL-6 protein is expressed in germinal-center B cells. Blood.

[B58] Falini B, Tiacci E, Pucciarini A, Bigerna B, Kurth J, Hatzivassiliou G, Droetto S, Galletti BV, Gambacorta M, Orazi A, Pasqualucci L, Miller I, Kuppers R, Dalla-Favera R, Cattoretti G (2003). Expression of the IRTA1 receptor identifies intraepithelial and subepithelial marginal zone B cells of the mucosa-associated lymphoid tissue (MALT). Blood.

[B59] Young LJT, Ip MM, Asch BB (2000). The cleared mammary fat pad and the transplantation of mammary gland morphological structures and cells. Methods in Mammary Gland Biology and Breast Cancer Research.

[B60] Rasmussen SB, Young LJT, Smith GH, Ip MM, Asch BB (2000). Preparing mammary gland whole mounts from mice. Methods in Mammary Gland Biology and Breast Cancer Research.

